# Subregional limbic radiomics on FDG-PET provides accurate early detection of Alzheimer’s disease

**DOI:** 10.1186/s12880-026-02168-8

**Published:** 2026-02-19

**Authors:** Ramin Rasi, Albert Guvenis, for the Alzheimer’s Disease Neuroimaging Initiative

**Affiliations:** 1 Faculty of Engineering and Natural Sciences, Department of Biomedical Engineering, Biruni University, Istanbul, 34015 Türkiye; 2 Biruni University Research Center (B@MER), Biruni University, Istanbul, 34015 Türkiye; 3 Institute of Biomedical Engineering, Boğaziçi University, Istanbul, Türkiye

**Keywords:** Alzheimer's disease (AD), FDG-PET, Radiomics, Hippocampus, Amygdala

## Abstract

**Background:**

To investigate the radiomics features of the hippocampus and the amygdala subregions in FDG-PET images that can best differentiate Mild Cognitive Impairment (MCI), Alzheimer’s Disease (AD), and healthy patients.

**Methods:**

Baseline FDG-PET data from 555 participants in the ADNI dataset were analyzed, comprising 189 cognitively normal (CN) individuals, 201 with MCI, and 165 with AD. We extracted 120 quantitative features from finely and automatically parcellated subregions (hippocampal *n* = 38, amygdala *n* = 18) using a probabilistic atlas. To identify the most effective classification model, we applied four feature selection techniques, ANOVA, PCA, LASSO, and Chi-square, combined with nine different classifiers, resulting in 36 unique model combinations. This comprehensive evaluation enabled the selection of a high-performing machine learning pipeline.

**Results:**

The Multi-Layer Perceptron (MLP) model combined with LASSO demonstrated excellent classification performance: ROC AUC of 0.957 for CN vs. AD, ROC AUC of 0.867 for MCI vs. AD, and ROC AUC of 0.782 for CN vs. MCI. Key regions, including the accessory basal nucleus, presubiculum head, and CA4 head, were identified as critical biomarkers. Features including GLRLM (Long Run Emphasis) and Small Dependence Emphasis (GLDM) showed strong diagnostic potential, reflecting subtle metabolic and microstructural changes often preceding anatomical alterations.

**Conclusions:**

Specific hippocampal and amygdala subregions and their four radiomic features were found to have a significant role in the early diagnosis of AD, its staging, and its severity assessment by capturing subtle shifts in metabolic patterns. Furthermore, these features offer potential insights into the disease’s underlying mechanisms and model interpretability.

**Supplementary Information:**

The online version contains supplementary material available at 10.1186/s12880-026-02168-8.

## Background

Alzheimer’s disease (AD), the most prevalent form of dementia, is a widespread condition globally, accounting for 60–70% of dementia cases, with about 10 million new incidences annually [1]. The development of AD is linked to complex neurobiological changes, including amyloid-beta plaque deposits, tau tangles, and inflammation, which have central roles in regulating cognitive deterioration [2]. Despite extensive efforts, the underlying mechanisms for the transition from mild cognitive impairment (MCI) to advanced dementia remain unknown [3]. A deeper understanding of such mechanisms, in particular by exploring less-studied brain regions and their metabolism, is crucial for the identification of new biomarkers and therapeutic targets.

AD diagnosis is routinely made based on a combination of cognitive testing, biomarker studies, and neuroimaging [3, 4]. Early diagnosis, especially in MCI, is challenging because MCI can or cannot progress to AD. It is thus critical to determine which instances of MCI are most likely to progress. Neuroimaging techniques, such as Fluorine-18-deoxyglucose positron emission tomography (FDG-PET), are very helpful in tracking changes in brain metabolism that are typical of neurodegeneration and provide early markers in MCI and AD [5, 6].

Neuroimaging research has particularly focused on the hippocampus and amygdala, interconnected structures that are severely affected by AD and are involved in memory and emotional processing [7, 8]. The hippo-amygdala complex also shows early atrophic and metabolic changes in AD and is therefore at the focus of disease onset research [9, 10]. The hippocampus, vulnerable in AD, shows volume losses associated with memory impairment. The amygdala, involved in emotional memory, also shows degeneration linked to emotional symptoms [11]. Degeneration in these regions can predict MCI progression to AD, and hence, they can be used as early diagnostic markers and therapeutic targets [12].

Recent studies have emphasized the diagnostic potential of hippocampal analysis in AD and MCI [13–15]. For example, MRI-based volumetry and functional connectivity analysis, and deep learning approaches, have reported promising performances in separating AD from normal controls, and, to a lesser extent, in separating MCI from AD and normal controls [16].

Radiomics, a computerized method that derives quantitative features from medical images, provides greater insight into brain alteration during the MCI-to-AD progression [17, 18]. Radiomics is especially powerful in assessing textural, morphological, and intensity-based features, and it can identify subtle changes that are not visible to the human eye, especially in PET and MRI images [19].

This strategy provides a comprehensive view of regionally specific texture and metabolic integrity, with a stress on heterogeneity that captures cellular or metabolic distinctions typical of AD [20–22]. While direct functional connectivity is typically measured using techniques like fMRI, our FDG-PET radiomics approach is highly informative because regional cerebral glucose metabolism rCMRglc is tightly coupled to synaptic activity and neuronal function [23]. Therefore, the hypometabolism characteristic of AD is a direct metabolic consequence of synaptic loss and compromised neuronal circuitry [24].

Our radiomic texture features, such as Small Dependence Emphasis (GLDM) and Long Run Emphasis (GLRLM), quantify the spatial heterogeneity and granularity of the FDG signal within a region, moving beyond simple mean uptake. This fine-grained variability reflects the cellular and microstructural disorganization, such as intermixed areas of preserved versus affected tissue, plaques, or tangles, that causes the disruption in connectivity [25]. Consequently, these textural features serve as a highly sensitive, quantitative surrogate marker for the microstructural substrate of disrupted neuronal connectivity, capturing subtle changes that often precede gross anatomical alterations [26].

While previous research has shown the importance of the hippocampus and amygdala for characterizing AD, substructures of these organs have not yet been, to our knowledge, examined by metabolic imaging. As FDG-PET imaging is more sensitive than structural imaging, and as metabolic changes precede anatomical changes [27, 28]. The present study aims at these regions, their subfields (e.g., CA1, CA3, subiculum), and nuclei specifically, which show differential vulnerabilities [29, 30]. We analyze radiomic features in these subregions of FDG-PET images for enhancing the detection of AD and its progression early.

The aim of this study is to recognize distinctive metabolic features within the subregions of the hippocampus and amygdala that distinguish between MCI, AD, and normal cognitive functioning with greater sensitivity, yielding an interpretable predictive model. By combining high-resolution subregional metabolic imaging with advanced quantitative radiomics and a rigorously optimized machine learning framework, we aim to provide a significantly more sensitive, accurate, and potentially earlier diagnostic tool for AD and MCI compared to existing approaches. Furthermore, we aim to produce a reduced set of imaging that may provide potential insights into the disease’s underlying mechanisms and model interpretability.

The novelty of our research lies in its unprecedented focus on the radiomic features within finely parcellated subregions of the hippocampus and amygdala on FDG-PET images. While the hippocampus and amygdala are known to be early targets in AD, previous studies have largely examined these structures at a macro level or primarily through structural MRI. Our approach is distinct because:

Subregional Granularity: We meticulously analyzed hippocampal and amygdala subregions, providing a far more granular view of metabolic changes than previously achieved. This fine-grained analysis may allow the detection of subtle, localized metabolic shifts that are indicative of early disease progression.

Radiomics on FDG-PET: We harnessed the power of radiomics, a quantitative imaging analysis method applied to FDG-PET, which captures metabolic activity. This combination enables the identification of intricate textural, morphological, and intensity-based features that are imperceptible to the human eye, offering deeper insights into the complex neurobiological changes associated with AD. Our findings confirm that these metabolic alterations precede gross anatomical changes, making them invaluable for early detection.

Robust Machine Learning Pipeline: We developed and rigorously evaluated a comprehensive machine learning pipeline, testing a large number of unique model combinations of feature selection techniques and classifiers on a large cohort of participants from the ADNI dataset.

## Methods

**Fig. 1:**
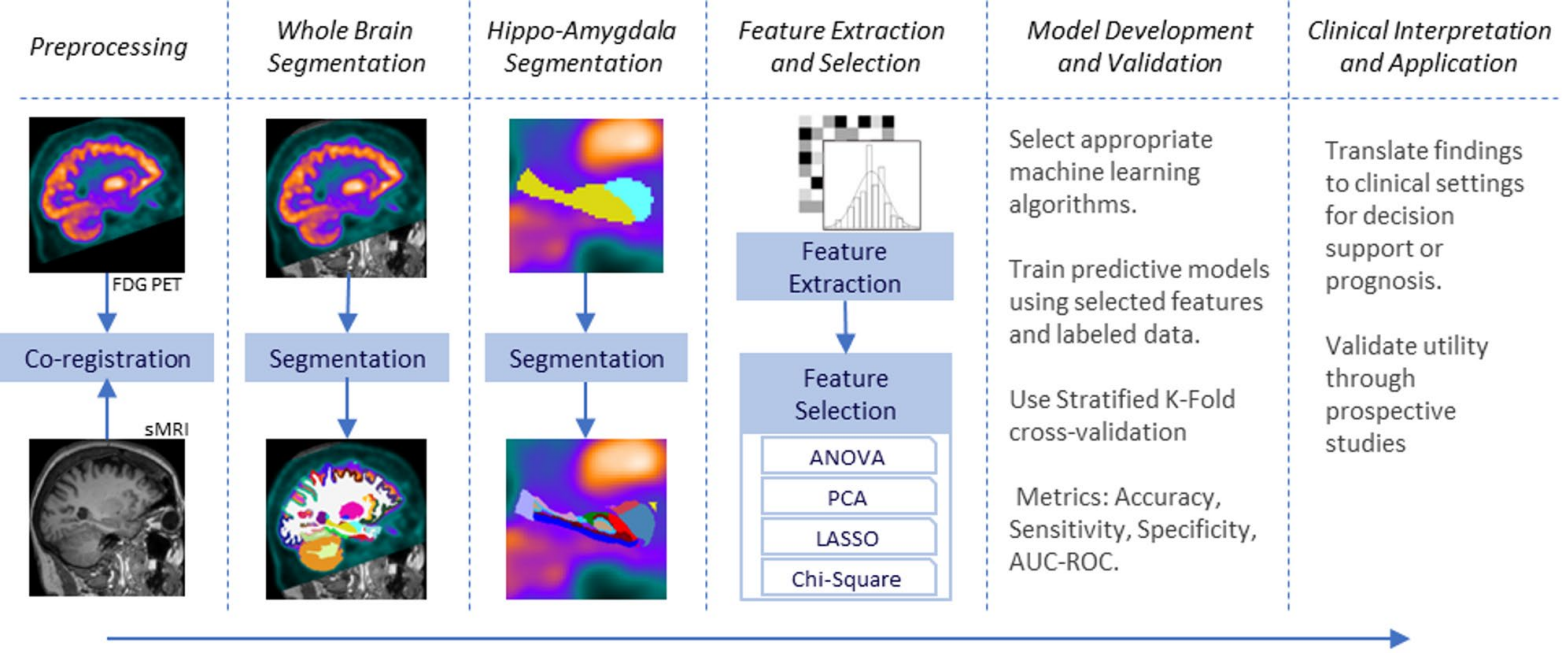
Pipeline for feature extraction and classification from hyppo-amygdala subregions

This study employed neuroimaging data from the Alzheimer’s Disease Neuroimaging Initiative (ADNI). After preprocessing and co-registering the FDG-PET and MRI images we segmented images into specific regions of interest. PyRadiomics was used to extract features from FDG-PET images, and dimensionality reduction techniques were applied to improve model performance. A diverse set of machine learning algorithms in combination with different feature selection methods were implemented and evaluated using stratified K-fold cross-validation. The outcomes were then refined and analyzed to support clinical decision-making, diagnosis, or prognosis. This approach enables the identification of informative biomarkers and the development of accurate diagnostic tools for early-stage cognitive impairment (Fig. 1).

### ADNI and participants

Data used in the preparation of this article were obtained from the Alzheimer’s Disease Neuroimaging Initiative (ADNI) database (adni.loni.usc.edu). The ADNI was launched in 2003 as a public-private partnership, led by Principal Investigator Michael W. Weiner, MD. The primary goal of ADNI has been to test whether serial magnetic resonance imaging (MRI), positron emission tomography (PET), other biological markers, and clinical and neuropsychological assessment can be combined to measure the progression of MCI and early Alzheimer’s disease. The data reveal a trend of decreasing cognitive function as measured by the Mini-Mental State Examination (MMSE) scores, with AD patients showing the lowest average score (23.1 ± 2.4) compared to MCI (27.5 ± 1.9) and CN (29.0 ± 1.2) groups, as shown in Table 1. This initial analysis suggests a potential link between clinical characteristics and disease progression, which our study aims to explore further using a radiomics approach.

**Table 1:** Clinical characteristics of participants

Clinical Diagnosis	No. Cases	Sex (M/F)	Age (mean ± SD)	MMSE (mean ± SD)
AD	165	93/72	74.6 ± 8.1	23.1 ± 2.4
MCI	201	121/80	72.7 ± 7.4	27.5 ± 1.9
CN	189	97/92	73.7 ± 6.3	29.0 ± 1.2
Total	555	306/249	73.6 ± 7.3	26.7 ± 3.1

### Co-registration

To accurately co-register FDG-PET and sMRI images, we utilized the rigid transformation feature of the ANTsX Tool. This method relies exclusively on rotational and translational adjustments, ensuring that the anatomical structures’ original shape and size remain unaltered. Rigid registration is particularly effective in scenarios where the images are only slightly misaligned in position or orientation, while their overall anatomical consistency is preserved. By employing this approach through ANTsX, we achieved precise and reliable co-registration of FDG-PET and sMRI data, facilitating seamless integration for subsequent analyses and interpretations [31].

### FDG-PET acquisition

This study used preprocessed FDG-PET data from the ADNI database. These comprehensive preprocessing steps, described in detail on the ADNI website (adni.loni.usc.edu), ensure uniformity across subjects, enabling robust comparative analyses. Dynamic 3D scans, acquired 30–60 min after administering 185 MBq (5 mCi) of radiotracer, consisted of six 5-minute frames. Preprocessed data underwent Co-Reg, AVG, Standardized Image, and Voxel Size adjustments, standardizing images to a 160 × 160 × 96 voxel grid with 1.5 mm isotropic resolution for consistent analyses [32].

### MRI acquisition

MRI data were collected following standardized protocols designed to maintain consistency across subjects and studies. Imaging was conducted on high-field MRI scanners (3T or higher), utilizing a range of pulse sequences such as T1-weighted, T2-weighted, fluid-attenuated inversion recovery (FLAIR), and diffusion tensor imaging (DTI). To reduce variability between scanners, uniform pulse sequences and standardized imaging parameters—including slice thickness, field of view, echo time, and repetition time—were carefully implemented [33].

### Segmentation

Segmentation of the hippocampus and amygdala into subfields and subnuclei was performed in two main steps using both broad anatomical mapping and detailed structural analysis. Initially, the entire brain was segmented into 95 regions of interest (ROIs) using the Desikan-Killiany-Tourville (DKT) atlas through the FastSurfer pipeline, leveraging its deep learning capabilities for rapid and efficient segmentation that provided a robust basis for detailed analyses [34]. Subsequently, using FreeSurfer, the hippocampus was divided into 19 subfields, and the amygdala into 9 subnuclei per hemisphere, employing probabilistic atlases and shape models to precisely measure volumes and define internal architectures [35–37]. Details of these segmented areas, identified by FS IDs, are presented in Table 2.

**Table 2:** IDs and corresponding subregions of the hippocampus and nuclei of the amygdala as segmented by FreeSurfer, detailing the hierarchical organization and anatomical substructures

Hippocampus			Amygdala	
FS ID	Subfields	substructures	FS ID	nuclei
203	parasubiculum	HEAD	7001	Lateral-nucleus
233	presubiculum-head		7003	Basal-nucleus
235	subiculum-head		7005	Central-nucleus
237	CA1-head		7006	Medial-nucleus
239	CA3-head		7007	Cortical-nucleus
241	CA4-head		7008	Accessory-Basal-nucleus
243	GC-ML-DG-head		7009	Corticoamygdaloid-transitio
245	molecular_layer_HP-head		7010	Anterior-amygdaloid-area-AAA
211	HATA		7015	Paralaminar-nucleus
234	presubiculum-body	BODY		
236	subiculum-body			
238	CA1-body			
240	CA3-body			
242	CA4-body			
244	GC-ML-DG-body			
246	molecular_layer_HP-body			
212	fimbria			
226	Hippocampal_tail	TAIL		
215	hippocampal-fissure	FISSURE		

### Feature extraction and selection

To extract meaningful features from FDG-PET images, the PyRadiomics package was utilized to compute 120 feature classes across 56 predefined regions of interest (ROIs). These features spanned various metrics, including first-order statistics, texture features such as GLCM, GLRLM, NGTDM, and GLSZM, as well as shape-based characteristics [38].

To address the curse of dimensionality and enhance model performance, we examined different dimension reduction methods. Several techniques were explored, including filter-based approaches like ANOVA and Chi-square, along with embedded methods such as the Least Absolute Shrinkage and Selection Operator (LASSO). Furthermore, Principal Component Analysis (PCA) was employed to reduce the dimensionality of the feature space while retaining critical information. This systematic selection process aimed to identify the most predictive features, ultimately strengthening the performance of the resulting models.

After implementing various feature selection and dimensionality reduction techniques, including ANOVA, Chi-square, LASSO, and PCA, we introduced an additional refinement step using Pearson Correlation Analysis to further enhance the feature set’s quality. This filter-based analysis is crucial for quantifying the linear relationships between the selected features, allowing us to systematically identify and eliminate redundant variables—typically those with a high absolute correlation (|r| > 0.9). By retaining only one feature from each highly correlated pair, this step successfully mitigated the issue of multicollinearity, ensuring that the final, refined feature set comprised maximally informative, non-redundant predictors, which is expected to significantly strengthen the robustness and predictive performance of the subsequent models.

### Classification

We implemented a diverse array of machine learning classifiers to achieve a reliable evaluation. The classification framework incorporated algorithms spanning multiple methodological categories, including ensemble methods (Gradient Boosting (GB), Random Forest (RF), AdaBoost (AB)), decision-based techniques (Decision Tree (DT)), probabilistic models (Gaussian Naive Bayes (GNB)), kernel-based approaches (Gaussian Process (GP)), neural networks (Multi-layer Perceptron (MLP)), discriminant analysis (Quadratic Discriminant Analysis (QDA), and proximity-based methods (K-Nearest Neighbors (KNN)) [39]. To ensure robust evaluation, we employed stratified K-fold cross-validation. We computed key metrics such as ROC AUC, accuracy, sensitivity, and specificity for each fold to assess the model’s performance. We tuned the hyperparameters for each classifier using a grid search approach. This involved exploring a range of values for key parameters such as learning rate, number of estimators, maximum depth, and others, as shown in Table 3.

**Table 3:** Values of the hyperparameters for the classifiers

Classifier	Parameter		
GB	learning_rate (0.01, 0.1, 0.2)	n_estimators (100, 200, 300)	max_depth (3, 5, 7)
HGB	learning_rate (0.01, 0.1, 0.2)	max_depth (3, 5, 7)	max_iter (100, 200, 300)
RF	n_estimators (100, 200, 300)	max_depth (None, 10, 20)	
DT	max_depth (3, 5, 10)		
GNB	var_smoothing (1e-9, 1e-8, 1e-7)		
MLP	hidden_layer_sizes: ((50,), (100,))	alpha ([0.0001, 0.001])	
QDA	n_estimators (50, 100, 200)	max_depth (None, 10, 20)	
KNN	n_neighbors (3, 5, 7)		

## Results

### Evaluation of classifiers and feature selection methods

Given the interdependence between feature selection methods and classifier performance, we evaluated multiple combinations of feature selection techniques (ANOVA, PCA, LASSO, Chi-square) and classifiers (GB, RF, AB, DT, GNB, GP, MLP, QDA, KNN) to enhance diagnostic accuracy for three groups: CN vs. AD, MCI vs. AD, and CN vs. MCI. After reducing features, we tested each subset with nine classifiers, optimizing them through grid search and validating with Stratified K-Fold cross-validation (k = 5) to maintain balanced data folds.

**Table 4:** Classification performance of different feature selection methods and classifiers for AD and MCI vs. CN

Comparison Groups	Feature Selection Method	Metric	AB	DT	GNB	GP	GB	HGB	MLP	QDA	RF
CN vs. AD	ANOVA	AUC	0.86	0.856	0.886	0.883	0.891	0.886	0.903	0.885	0.896
		ACC	0.771	0.788	0.811	0.797	0.802	0.78	0.828	0.802	0.808
	PCA	AUC	0.863	0.861	0.879	0.882	0.901	0.901	0.892	0.878	0.905
		ACC	0.794	0.791	0.797	0.791	0.825	0.831	0.811	0.8	0.831
	LASSO	AUC	0.939	0.851	0.95	0.906	0.931	0.94	0.957	0.911	0.937
		ACC	0.859	0.799	0.89	0.856	0.876	0.898	0.901	0.839	0.879
	Chi-square	AUC	0.869	0.864	0.889	0.868	0.893	0.89	0.889	0.883	0.891
		ACC	0.794	0.802	0.814	0.771	0.816	0.814	0.802	0.814	0.819
MCI vs. AD	ANOVA	AUC	0.763	0.773	0.816	0.8	0.796	0.803	0.811	0.799	0.774
		ACC	0.691	0.727	0.754	0.746	0.73	0.741	0.751	0.743	0.708
	PCA	AUC	0.739	0.744	0.778	0.778	0.79	0.778	0.782	0.778	0.777
		ACC	0.686	0.664	0.7	0.694	0.719	0.708	0.716	0.7	0.727
	LASSO	AUC	0.827	0.774	0.871	0.817	0.856	0.86	0.867	0.812	0.861
		ACC	0.757	0.729	0.792	0.787	0.771	0.776	0.77	0.746	0.798
	Chi-square	AUC	0.754	0.787	0.812	0.763	0.806	0.795	0.793	0.807	0.791
		ACC	0.708	0.732	0.568	0.713	0.735	0.721	0.735	0.711	0.711
CN vs. MCI	ANOVA	AUC	0.674	0.678	0.721	0.691	0.729	0.735	0.719	0.662	0.728
		ACC	0.615	0.651	0.659	0.659	0.682	0.669	0.664	0.621	0.667
	PCA	AUC	0.665	0.601	0.703	0.595	0.672	0.663	0.714	0.675	0.689
		ACC	0.633	0.585	0.641	0.582	0.638	0.61	0.656	0.605	0.638
	LASSO	AUC	0.762	0.68	0.792	0.751	0.788	0.787	0.803	0.746	0.796
		ACC	0.7	0.641	0.674	0.708	0.723	0.732	0.731	0.685	0.741
	Chi-square	AUC	0.631	0.639	0.693	0.652	0.684	0.682	0.698	0.637	0.692
		ACC	0.595	0.595	0.572	0.621	0.623	0.633	0.618	0.531	0.626

The performance of the models was assessed by ROC AUC and accuracy metrics. The combination of the MLP classifier with LASSO feature selection emerged as the most effective, achieving an ROC AUC of 0.957 and an accuracy of 0.901 for CN vs. AD, an AUC of 0.867 and an accuracy of 0.77 for MCI vs. AD, and an AUC of 0.803 and an accuracy of 0.731 for CN vs. MCI, as detailed in Table 4. Further analysis was conducted on the top-performing models to identify the key radiomic features and subregions driving the predictive performance, particularly focusing on the differences between CN, AD, and MCI within the hippocampal and amygdala subregions.

### Classification performance and clinical utility

Three pairwise binary classifiers were trained using all non-zero LASSO-selected radiomic features from hippocampal and amygdala subregions, followed by MLP classification and isotonic probability calibration. Final models were evaluated on an independent 30% hold-out test set that was never used during feature selection or model training.

**Fig. 2:**
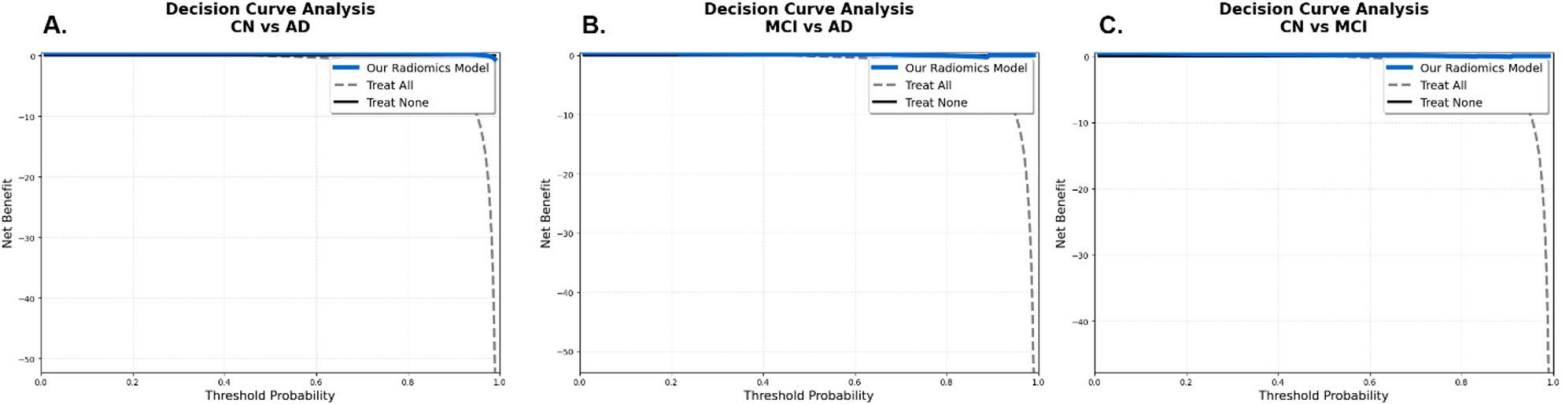
Decision curve analysis (DCA) demonstrating the clinical utility of the subregional hippocampal and amygdala radiomics signature across three diagnostic comparisons on the independent test set. (**A**) CN vs. AD (**B**) MCI vs. AD (**C**) CN vs. MCI

Performance metrics and decision curve analysis on the independent test set are summarized in Table 5; Fig. 2.

**Table 5:** Final model performance on the independent 30% hold-out test set

Comparison	*N* features	ROC AUC	Accuracy	Sensitivity	Specificity	Max Net Benefit (DCA)
CN vs. AD	36	0.955	0.935	0.9	0.965	0.455
MCI vs. AD	33	0.862	0.782	0.78	0.783	0.44
CN vs. MCI	35	0.757	0.701	0.6	0.807	0.508

Decision curve analysis confirmed substantial clinical utility across all diagnostic tasks (Fig. 2). The radiomics signature consistently outperformed both “treat all” and “treat none” strategies, yielding high net benefit (0.44–0.51) over a wide range of clinically plausible risk thresholds. Notably, the CN vs. MCI comparison, representing the earliest detectable stage of cognitive decline, achieved the highest maximum net benefit (0.508), highlighting the model’s particular value for preclinical screening and risk stratification.

All detailed feature lists, LASSO rankings, and calibration diagnostics are provided in Supplementary File S2.

### Analysis of key features and regions

To ensure the stability and reliability of texture features, we examined the volumetric characteristics of all segmented hippocampal and amygdala subregions. Supplementary Table S1 lists the average volume (mm³) and voxel count for each of the 56 subregions. IN this stage, subregions corresponding to the medial nucleus (FS ID 7006) and cortical nucleus (FS ID 7007) containing fewer than 25 voxels (< 84.4 mm³) were excluded from radiomic feature extraction due to the instability of high-order texture metrics in very small ROIs. No artificial merging of adjacent areas was performed, ensuring that the segmentation preserved anatomical fidelity throughout the analysis pipeline. Then, our in-depth analysis of uncorrelated features for both diagnosis groups identified two key features as the common subset for both groups, CN vs. AD and MCI vs. AD: Long Run Emphasis (GLRLM) in the left accessory basal nucleus and Small Dependence Emphasis (GLDM) in the left presubiculum-head. These features were found to be the most effective in differentiating between MCI, CN, and AD individuals among the highly uncorrelated feature pairs.

**Table 6:** Performance evaluation of the four most effective features individually and in combination for classifying CN vs. AD, MCI vs. AD, and CN vs. MCI using MLP

	Uncorrelated Features	ROC AUC ± SD	Accuracy ± SD	Sensitivity	Specificity
CN vs. AD	original_gldm_SmallDependenceEmphasis_233_lh	0.782 ± 0.008	0.723 ± 0.016	0.618	0.815
	original_glrlm_LongRunEmphasis_7008_lh	0.885 ± 0.020	0.797 ± 0.039	0.739	0.847
	original_gldm_SmallDependenceEmphasis_233_lh	0.903 ± 0.019	0.822 ± 0.049	0.776	0.863
	& original_glrlm_LongRunEmphasis_7008_lh				
MCI vs. AD	original_gldm_SmallDependenceEmphasis_233_lh	0.748 ± 0.052	0.686 ± 0.051	0.558	0.791
	original_glrlm_LongRunEmphasis_7008_lh	0.798 ± 0.025	0.735 ± 0.035	0.691	0.771
	original_gldm_SmallDependenceEmphasis_233_lh	0.824 ± 0.026	0.749 ± 0.040	0.715	0.776
	& original_glrlm_LongRunEmphasis_7008_lh				
CN vs. MCI	original_firstorder_RobustMeanAbsoluteDeviation_7008_lh	0.631 ± 0.072	0.62 ± 0.038	0.611	0.629
	original_firstorder_Minimum_241_lh	0.631 ± 0.032	0.613 ± 0.014	0.607	0.619
	original_firstorder_RobustMeanAbsoluteDeviation_7008_lh	0.677 ± 0.059	0.636 ± 0.076	0.606	0.666
	& original_firstorder_Minimum_241_lh				

These features achieved remarkable performance in classification tasks using the MLP classifier. For CN vs. AD, it yielded an AUC of 0.903, accuracy of 0.822, sensitivity of 0.863, and specificity of 0.776. In MCI vs. AD classification, the same classifier achieved an AUC of 0.824, accuracy of 0.749, sensitivity of 0.776, and specificity of 0.715 (Table 6). For CN vs. MCI, Robust Mean Absolute Deviation (First order) and Minimum (First order) were the most effective uncorrelated features, achieving an AUC of 0.677 and an accuracy of 0.636 when combined.

**Fig. 3:**
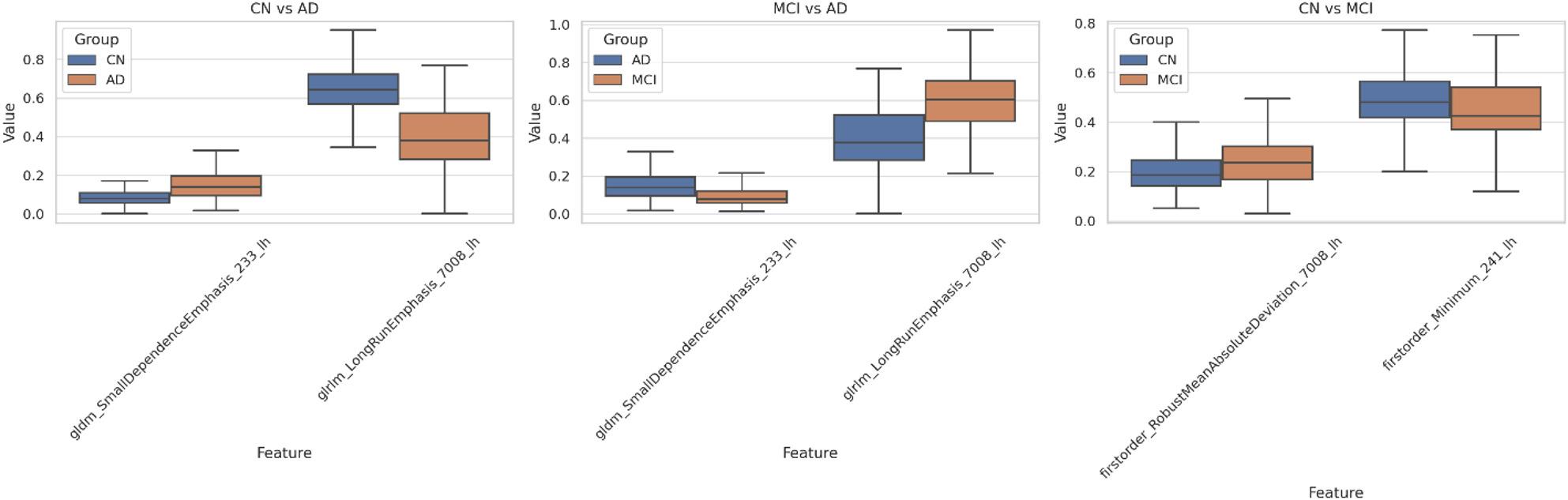
Comparison of radiomic features across diagnostic groups: Boxplots illustrate the normalized values of selected radiomic features for three key comparisons: CN vs. AD, MCI vs. AD, and CN vs. MCI

Table 7; Fig. 3 highlight structural and textural changes in the hippocampal-amygdala complex across CN, MCI, and AD, emphasizing specific radiomic features as biomarkers. For CN vs. AD, Small Dependence Emphasis (GLDM) shows a notable increase in mean values (0.0839 to 0.1636), indicating greater gray-level heterogeneity, while Long Run Emphasis (GLRLM) decreases significantly (0.6429 to 0.3988), reflecting reduced structural uniformity. Both features have highly significant p-values, underscoring their diagnostic relevance.

A similar trend can be seen in MCI vs. AD, where Small Dependence Emphasis (GLDM) increases from 0.0929 in MCI to 0.1636 in AD, and glrlm_LongRunEmphasis decreases from 0.5912 to 0.3988, reinforcing these features as able to tell AD from earlier stages. The changes observed in CN vs. MCI are subtler. Robust Mean Absolute Deviation (First order), for example, increases from 0.2042 to 0.2453, indicative of higher variability in the texture in MCI, and the Minimum (First order) decreased from 0.4864 to 0.4563, indicating a loss of signal intensity. This trend is underlined by their respective p-values of 0.0001 and 0.0325.

**Table 7:** Comparison of radiomic features across CN, MCI, and AD groups, highlighting mean, standard deviation, and statistical significance

Comparison	Feature	Group1_Mean	Group2_Mean	Group1_Std	Group2_Std	P_Value
CN vs. AD	original_gldm_SmallDependenceEmphasis_233_lh	0.0839	0.1636	0.0423	0.1193	0.001
	original_glrlm_LongRunEmphasis _7008_lh	0.6429	0.3988	0.1223	0.1561	0.001
MCI vs. AD	original_gldm_SmallDependenceEmphasis_233_lh	0.0929	0.1636	0.0536	0.1193	0.001
	original_glrlm_LongRunEmphasis _7008_lh	0.5912	0.3988	0.1659	0.1561	0.001
CN vs. MCI	original_firstorder_RobustMeanAbsoluteDeviation_7008_lh	0.2042	0.2453	0.0941	0.1134	0.001
	original_firstorder_Minimum _241_lh	0.4864	0.4563	0.1238	0.1511	0.0325

### Case study: Longitudinal analysis of radiomic features in AD progression

#### Patient overview

As a case study, we examined the long-term data of a single patient from the ADNI database. Over eight years, the patient was subjected to various scans and clinical assessments, transitioning from CN to AD. We selected four FDG-PET scans that illustrate this progression. Additionally, clinical evaluations were recorded, and scores involving MMSE, ADAS11, ADAS13, and CDRSB scores were presented in Table 8.

**Table 8:** Radiomic features and clinical scores over time

Study Date	MMSE	ADAS11	ADAS13	CDRSB	Original_gldm_ SmallDependenceEmphasis_ (presubiculum-head_lh)	Original_glrlm_ LongRunEmphasis_ (Accessory-Basal-nucleus_lh)	Original_ firstorder_Robust MeanAbsoluteDeviation_ (Accessory-Basal-nucleus_lh)	Original_ firstorder_Minimum_ (CA4-head_lh)
4/11/2006	29	8	11	0	0.077763	0.576802	0.086043	0.415374
4/8/2008	27	13.33	18.33	2.5	0.352112	0.266491	0.125388	0.432891
1/20/2012	22	17	27	7	0.569209	0.207079	0.178754	0.456195
1/28/2014	18	33	46	10	0.688984	0.134096	0.203053	0.705742

#### Methodology

Radiomic features were extracted from the three subregions, including the accessory basal nucleus, presubiculum head, and CA4 head, using PyRadiomics. Key features, namely, Small Dependence Emphasis (GLDM), Long Run Emphasis (GLRLM), Robust Mean Absolute Deviation (First order), and Minimum (First order) were computed and analyzed across the four scans.

#### Results of the case study

**Fig. 4:**
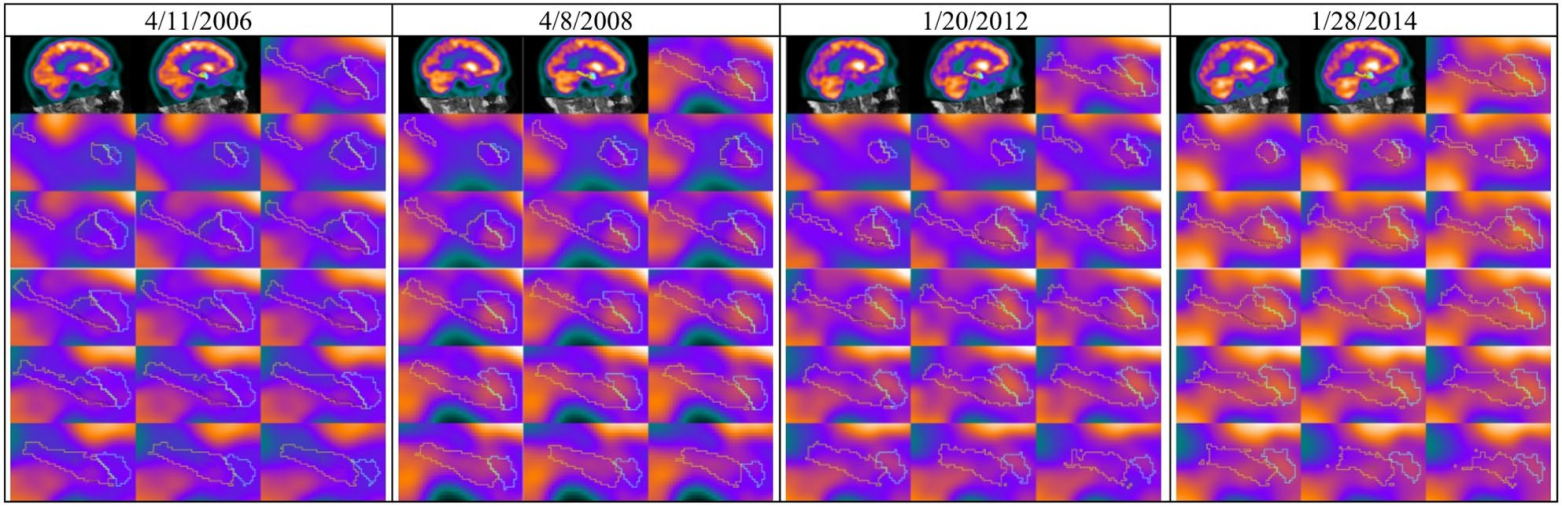
Longitudinal FDG-PET scans with segmented  hippocampal and  amygdala subregions ( accessory basal nucleus, Presubiculum,  CA4), demonstrating progressive changes in key areas

The patient exhibited a steady decline in clinical scores (MMSE from 29 to 18 and CDRSB from 0 to 10), correlating with significant changes in radiomic features. The temporal evolution of each radiomic feature is shown in Table 8 and visualized in Fig. 4, highlighting trends and abrupt shifts in metabolic patterns. The possible interpretations of observed changes in these computed radiomics features for the above-mentioned specific three sub-regions of the hippocampal-amygdala complex are given in the next section.

## Discussion

Existing radiomic schemes utilizing FDG-PET scans achieve high accuracy in differentiating AD, MCI, and CN participants. However, these voxel- or region-of-interest (ROI) based approaches often lack the fine-grained anatomical and biological specificity needed for clinical interpretability. This study addresses this crucial gap by, for the first time, investigating radiomic features within the highly detailed subregions of the hippocampus and amygdala using baseline FDG-PET scans from 555 participants in the ADNI database. We segmented the hippocampus and amygdala into 38 subfields and 18 subnuclei using probabilistic atlases. We then extracted 120 radiomic features for each segmented subfield and subnucleus using the PyRadiomics tool. By combining various machine learning classifiers and feature selection techniques, we identified the most relevant features for predicting the progression of Alzheimer’s disease. Ultimately, analysis of these features led to a reduction in complexity, resulting in a more interpretable few features.

The findings indicate that these radiomic features not only serve as effective diagnostic tools but also offer promise as imaging metabolic biomarkers for early detection and monitoring of AD progression, as well as for formulating new hypotheses about the biological mechanism of the disease and defining specific therapeutic targets.

**Table 9:** Summary of imaging techniques and methodologies for classifying alzheimer’s disease stages based on hippocampal and amygdala features

DOI / Ref	Methodology	Regions of Interest	Imaging Modality	Dataset Size	Performance Metrics
[16]	Volumetric analysis using automated segmentation	Hippocampal and Amygdala Subfields	MRI	92	AD vs. NC: AUC 0.97 MCI vs. NC: AUC 0.79 AD vs. MCI: AUC 0.81
[15]	Functional connectivity analysis with support vector machine learning	Hippocampus	fMRI	119	AD vs. NC: Acc 0.82 MCI vs. NC: Acc 0.81 AD vs. MCI: Acc 0.81
[13]	3D deep convolutional neural network incorporating visual and global shape features	Hippocampus	MRI	933	AD vs. CN: AUC 0.978
[14]	Segmentation and volumetric analysis	Hippocampus	MRI	373	AD vs. CN: Acc 0.944
Proposed Method	Radiomic feature extraction with Multi-layer Perceptron classifier analysis	Hippocampal and Amygdala Subfields	FDG PET	555	AD vs. NC: AUC 0.957 MCI vs. NC: AUC 0.782 AD vs. MCI: AUC 0.867

The previous studies summarized in Table 9 explore various imaging and classification techniques that have been used to predict Alzheimer’s disease stages, focusing on the hippocampus and amygdala. Our work is the first study employing FDG PET, known for its sensitivity and potential for earlier diagnosis [40]. The key contributions of our study can be summarized as (i) the use of FDG PET imaging, (ii) a larger patient sample, and (iii) a limited set of radiomic features enhancing both explainability and interpretability.

By concentrating on metabolic data, the study reveals early biochemical changes linked to Alzheimer’s disease, capturing subtle disease progressions that structural imaging might overlook. With a robust sample size of 555 cases, the findings offer strong generalizability and reliability. Therefore, our approach provides a framework for both the early identification of Alzheimer’s disease and the differentiation of MCI from AD or normal controls, making it a potential method for clinical and research applications.

Four significant radiomic features from FDG-PET scans distinguished between MCI, AD, and CN states, capturing subtle gray-level variations indicative of underlying AD pathology. Only two of them are used for distinguishing specific AD stages.

- Small Dependence Emphasis (SDE): Measures the prevalence of small gray-level dependencies. A high SDE means lots of subtle variations in gray levels.
- Long Run Emphasis (LRE): Measures the prevalence of long runs of consecutive pixels with the same gray level. A low LRE means fewer long runs and more short runs, suggesting a less uniform or more rapidly changing texture.
- Original_firstorder_Robust_MeanAbsoluteDeviation: This radiomics feature measures the variability of pixel/voxel intensities within a region of interest in the image. It does so by calculating the mean absolute deviation, but in a “robust” way to minimize the impact of any unusually high or low-intensity values that might be present.
- An increasing Original_firstorder_Robust_MeanAbsoluteDeviation in a brain region suggests a trend towards greater diversity in tissue characteristics, which could be related to disease progression, treatment response, or other factors. In conditions like Alzheimer’s disease, there might be a mix of healthy tissue, atrophied tissue, and areas with plaques or tangles, contributing to increased heterogeneity. Note that successful treatment may also initially increase heterogeneity as the tissue undergoes changes (e.g., inflammation, edema) before eventually returning to a more normal state.
- Original_firstorder_Minimum: This radiomics feature identifies the single lowest intensity value within a defined area of the image. An increase suggests that the lowest glucose uptake values within a region have become higher. This could indicate improved tissue health, increased neuronal activity, or other metabolic changes, such as inflammation, when the disease progresses.

The high predictive power of the identified radiomic features is rooted in the known pathological mechanisms of Alzheimer’s Disease (AD), providing a quantitative link between image texture and microstructural tissue disruption [41]. The texture-based features, derived from GLRLM and GLSZM, act as sensitive biomarkers of the heterogeneous metabolic landscape caused by neurodegeneration. Specifically, the significant increase observed in Small Dependence Emphasis (SDE), which measures the homogeneity of small dependence regions, directly reflects the profound gray-level heterogeneity characteristic of AD-related hypometabolism on FDG-PET. This spatial heterogeneity is a complex signal driven by the co-existence of metabolically suppressed tissue, areas of pronounced neuronal loss (atrophy), and localized inflammatory or amyloid/tau plaque burdens, which create a ‘patchwork’ of differing glucose uptake levels. Conversely, the substantial decrease in Long Run Emphasis (LRE), which typically signifies large, uniform areas of gray level, points to a reduction in the structural uniformity and organization of the tissue. This loss of textural consistency strongly correlates with widespread synaptic loss and progressive neuronal degeneration. As healthy, organized neuronal tissue is replaced by atrophic or pathologically altered tissue, the spatial extent of uniform glucose uptake regions diminishes, thereby reducing LRE. Furthermore, the prominence of the accessory basal nucleus, presubiculum head, and CA4 head as highly predictive subregions is mechanistically critical and anatomically aligned with AD’s trajectory [42]. The accessory basal nucleus, a component of the amygdala, is intimately involved in emotional memory and its vulnerability in AD, providing an anatomical context for why its radiomic features are so highly predictive [43, 44]. The presubiculum and CA4 regions of the hippocampus are similarly vulnerable, serving as crucial hubs in the Papez circuit that display early signs of hypometabolism and atrophy. By synthesizing these findings, our radiomic analysis provides a data-driven window into the microstructural and metabolic breakdown underpinning AD pathogenesis within the most vulnerable limbic structures.

Note that, as expressed above, the changes in radiomics features seen for the case study summarized in Table 8 may indicate a mix of healthy tissue, atrophied tissue, and areas with plaques or tangles, contributing to increased heterogeneity as well as inflammation, which should not be confused with increased metabolism.

Our findings align with existing theories about Alzheimer’s pathology, supporting the view that the hippocampus and amygdala are early targets of neurodegeneration in AD [42, 45]. The distinct textural changes captured by the identified features provide a nuanced characterization of these regions, indicating alterations in gray-level uniformity and structural heterogeneity. By highlighting specific subregions such as the accessory basal nucleus, presubiculum head, and CA4-head, this study demonstrates the potential of radiomics to reveal subtle but clinically significant changes in Alzheimer’s disease progression. These changes correlate with known neurodegenerative processes, including atrophy and disrupted neuronal connectivity [41]. Furthermore, the study goes beyond traditional volumetric measures, offering a more detailed characterization and understanding of these brain regions and their involvement in Alzheimer’s pathology.

The findings in Table 6 emphasize that the accessory basal nucleus may be an important subregion among all subregions of the hippocampal-amygdala complex for all states of AD. These results highlight the potential importance of the accessory basal nucleus, a subnucleus of the amygdala, in the pathology of Alzheimer’s disease. Its pronounced ability to differentiate diagnostic categories suggests it may play a critical role in early disease mechanisms. Further exploration of the neurobiological factors behind this observation is warranted, as it could deepen our understanding of Alzheimer’s progression, particularly regarding functional changes in the amygdala. The findings in this study confirm the important knowledge about metabolic changes in the hippocampal-amygdala complex in Alzheimer’s disease.

Our evaluation framework meticulously examined the robustness and performance of the model through a structured analysis involving nine classifiers combined with four feature selection techniques, resulting in 36 distinct configurations. Each configuration underwent rigorous evaluation based on two key metrics—accuracy (ACC) and area under the ROC curve (AUC)—to comprehensively assess predictive performance. This detailed approach highlights the reliability and adaptability of our methodology, culminating in the selection of the MLP classifier coupled with LASSO for feature selection. To ensure unbiased results, stratified k-fold cross-validation (k = 5) was implemented. The selected models exhibited strong performance in distinguishing between CN and AD (ROC AUC: 0.957, Accuracy: 0.907), MCI and AD (ROC AUC: 0.867, Accuracy: 0.806), and CN and MCI (ROC AUC: 0.782, Accuracy: 0.753).

We expect the important impacts of these findings on clinical practice and AD research.

- Exceptional Diagnostic Accuracy: The model achieved outstanding classification performance, with ROC AUCs of 0.957 for differentiating CN vs. AD, 0.867 for MCI vs. AD, and 0.803 for CN vs. MCI. The strong performance in distinguishing MCI from AD, in particular, represents a significant step forward in identifying individuals at the highest risk of progression.
- Identification of Critical Biomarkers: The work successfully identified specific subregions, such as the accessory basal nucleus, presubiculum head, and CA4 head, as critical biomarkers. Furthermore, features like GLRLM (Long Run Emphasis) and Small Dependence Emphasis (GLDM) proved to have strong diagnostic potential, reflecting underlying metabolic and microstructural changes.
- Enhanced Interpretability: The rigorous feature selection and correlation analyses resulted in a refined set of highly uncorrelated features, leading to a more interpretable predictive model. This clarity can foster greater clinical adoption and facilitate a deeper understanding of the disease’s underlying mechanisms.

There are, however, several limitations to our study. Firstly, the cross-sectional design of the FDG-PET data limits the assessment of longitudinal trends crucial for understanding disease progression [46]. Consequently, the inclusion of a single longitudinal case study, while valuable as an illustrative proof-of-concept to demonstrate the dynamic changes in our identified features during progression, lacks the statistical power and sample size required to establish population-level robustness or definitively assess the long-term predictive stability of the radiomic signature. Furthermore, a comprehensive feature stability analysis, such as Intraclass Correlation Coefficient (ICC) or test-retest reproducibility, was not included. This crucial validation step was constrained by the limited availability of FDG-PET test-retest imaging pairs within the ADNI cohort, which prevented a statistically robust assessment of the stability of our sub-regional radiomic features. Secondly, the generalizability of our findings may be limited by the specific characteristics of the ADNI cohort. To enhance robustness and applicability, increasing the diversity and number of populations and databases for data extraction is necessary [47]. Thirdly, despite our rigorous internal validation using stratified K-fold cross-validation, the current study lacks independent external validation on a distinct patient cohort, which is essential for definitively establishing the model’s generalizability and reliability across different patient demographics and imaging protocols. Finally, and critically for full clinical adoption, while we have demonstrated the net clinical benefit of our model via Decision Curve Analysis (DCA), a comprehensive analysis of the model’s calibration is currently absent. Specifically, we did not present the Hosmer-Lemeshow (HL) Test or Calibration Curves. While our metrics (e.g., AUC, accuracy, and DCA) demonstrate discrimination and utility, the omission of these specific analyses means the model’s performance in terms of accurate risk stratification (calibration) remains unquantified. These necessary calibration steps will be the primary focus of our future work, alongside external validation, to ensure our findings translate into a trustworthy and practical clinical tool. Additionally, while feature reduction improved biomarker interpretability, it may have excluded potentially relevant features, especially those indicating subtle but critical changes in other brain regions. This underscores the need for careful consideration of meaningful correlations during feature selection to avoid omitting important information. These results warrant further investigation and validation in independent datasets, integrating radiomic biomarkers with clinical and genetic data to refine predictive models. We acknowledge that despite harmonization strategies (resampling, atlas-based co-registration), partial-volume effects may still influence quantitative estimates, especially in the small subregions. The current approaches partly alleviate this limitation but cannot replace a full mathematical PVE correction. Therefore, future longitudinal work should incorporate explicit partial volume correction algorithms to refine metabolic quantification. Addressing standardization in image acquisition, feature extraction, and analysis is crucial for clinical translation. Moreover, larger, multicenter studies are needed to confirm these findings and develop robust clinical decision-support systems [48].

## Conclusions

In conclusion, this study highlights the promise of FDG PET radiomic biomarkers of specific subregions for the early diagnosis and staging of Alzheimer’s disease using a streamlined radiomic approach. By focusing on the hippocampus and amygdala, and using only four features, new insights can be generated into the disease’s metabolic underpinnings. Using a low number of features also helps the interpretability of this scheme. Future efforts should concentrate on longitudinal validation and real-world clinical translation, as well as investigating the effects of tailored targeted therapies on the discovered biomarkers and refining further their biological interpretation.

## Supplementary Information

Below is the link to the electronic supplementary material.

Supplementary Material 1

Supplementary Material 2

## Data Availability

The datasets analyzed during the study are publicly available and can be accessed through the Alzheimer’s Disease Neuroimaging Initiative (ADNI) repository at [https://adni.loni.usc.edu].

## Abbreviations

AB: AdaBoost
AD: Alzheimer’s disease
ADNI: Alzheimer’s disease neuroimaging initiative
ANOVA: Analysis of variance
AUC: Area under the curve
CN: Cognitively normal
DCA: Decision curve analysis
DKT: Desikan-Killiany-Tourville
DT: Decision Tree
DTI: Diffusion tensor imaging
FDG-PET: Fluorine-18-deoxyglucose positron emission tomography
FLAIR: Fluid-attenuated inversion recovery
FS ID: FreeSurfer ID
GB: Gradient boosting
GC-ML-DG: Granular cell and molecular layer of the dentate gyrus
GLCM: Gray level co-occurrence matrix
GLDM: Gray level dependence matrix
GLRLM: Gray level run length matrix
GLSZM: Gray level size zone matrix
GNB: Gaussian naive bayes
GP: Gaussian process
KNN: K-nearest neighbors
LASSO: Least absolute shrinkage and selection operator
MCI: Mild cognitive impairment
MLP: Multi-layer perceptron
MMSE: Mini-mental state examination
MRI: Magnetic resonance imaging
PCA: Principal component analysis
PET: Positron emission tomography
QDA: Quadratic discriminant analysis
RF: Random forest
ROIs: Regions of interest
ROC AUC: Receiver operating characteristic area under the curve
sMRI: Structural magnetic resonance imaging
