# Supplementary material for: Subregional limbic radiomics on FDG-PET provides accurate early detection of Alzheimer’s disease
Source: BMC Med Imaging. 2026 Feb 19;26:98. doi: 10.1186/s12880-026-02168-8 (PMC12922427; doi:10.1186/s12880-026-02168-8)
Supplement: Supplementary file 2 — Supplementary Material 2 [file 12880_2026_2168_MOESM2_ESM.docx]

## CN vs. AD

In the comparative analysis for CN vs. AD, we used the MLP classifier and a rank-based approach to identify the most predictive subset from the top 50 features with the largest absolute LASSO coefficients, reflecting the most informative non-zero weights from the regression model.

Features were ranked in descending order of their absolute LASSO coefficients and added one by one to the feature set. For each subset size, an MLP model was trained using stratified cross-validation, and its performance was evaluated. This iterative procedure enabled systematic identification of the optimal subset.

As shown in Figure 2, the optimal subset comprised 36 features, where the MLP classifier demonstrated outstanding performance, achieving a ROC AUC of 0.951, an accuracy of 0.907, a specificity of 0.916, and a sensitivity of 0.897. This configuration was selected as the optimal point based on the average of ROC AUC and accuracy, emphasizing the significance of these metrics in evaluating prediction models. These results demonstrate the effectiveness of this subset in differentiating between AD and CN subjects.


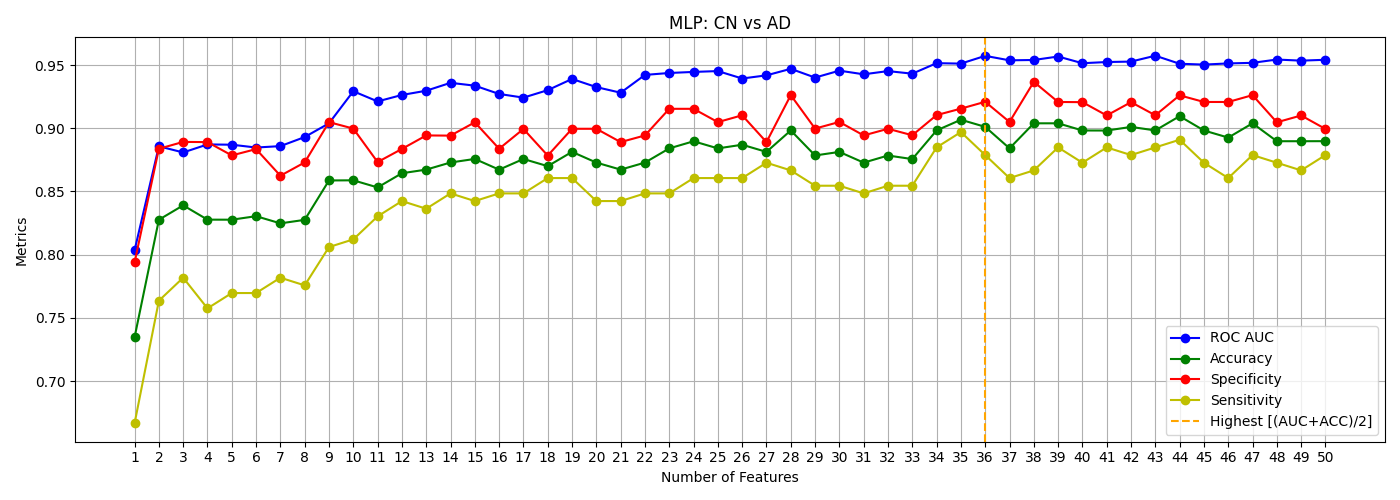


**Fig. 2.** Performance of MLP Classifier with LASSO-Selected Features for CN vs. AD Discrimination


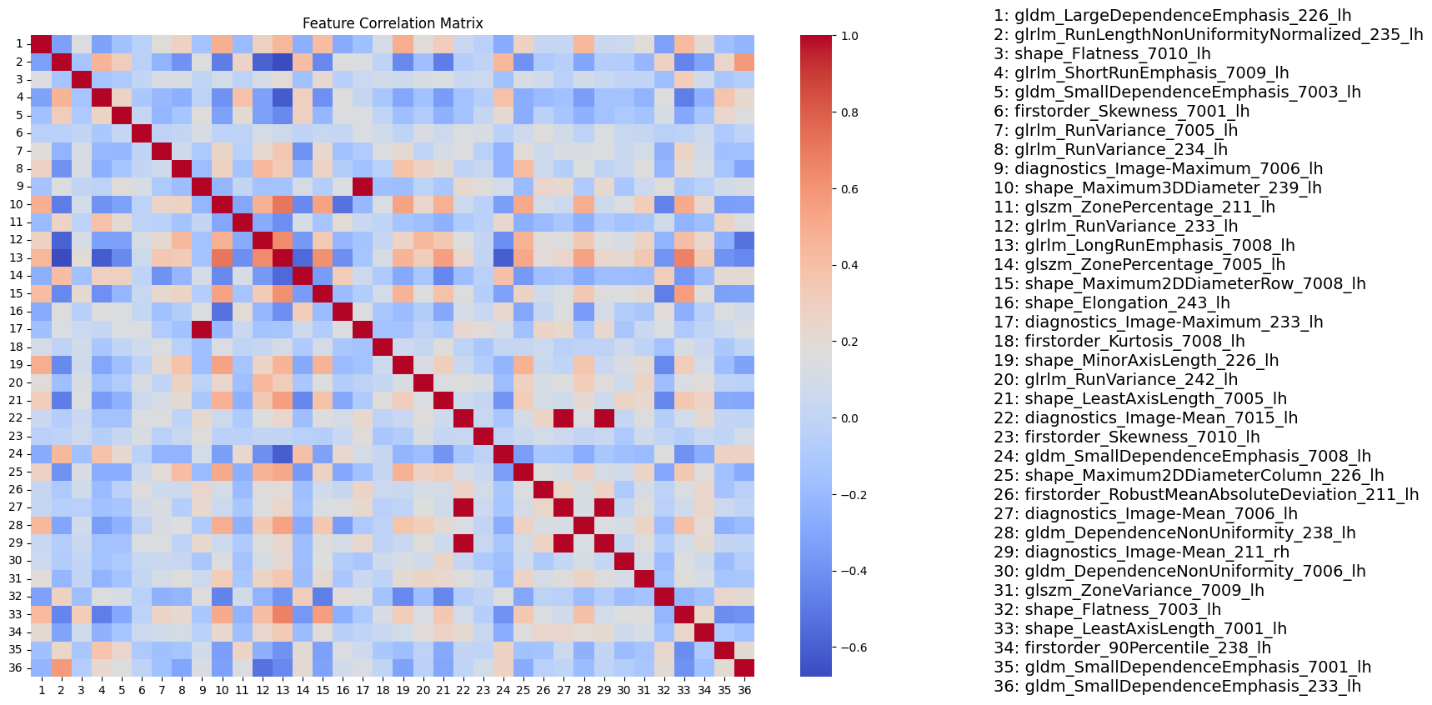


**Fig. 3.** Feature correlation matrix for the group of features with the highest accuracy in discriminating CN from AD

Further refinement involved Pearson correlation analysis on these 36 features (Figure 3) to reduce redundancy and ensure each feature contributed uniquely to the model.

Features with high pairwise correlation were excluded to reduce multicollinearity, and selection of uncorrelated features was supported using Spearman rank correlation to confirm the absence of statistically significant associations. The selected features, detailed in Table 5, exhibit minimal correlation, establishing a robust foundation for the predictive model.

**Table 5:** Highly uncorrelated feature pairs for distinguishing CN vs. AD identified through the Pearson correlation test, showing minimal correlation and corresponding p-values

| Uncorrelated Features (Feature Name, FS ID, left/right hand) | | Correlation | P-Value |
| --- | --- | --- | --- |
| original_shape_Flatness_7003_lh | original_firstorder_Skewness_7010_lh | 0.001 | 8.64E-01 |
| original_firstorder_Kurtosis_7008_lh | original_gldm_SmallDependenceEmphasis_233_lh | 0.002 | 8.93E-01 |
| original_firstorder_Skewness_7010_lh | original_glrlm_LongRunEmphasis_7008_lh | 0.003 | 6.58E-01 |
| original_glrlm_RunVariance_242_lh | original_glrlm_RunVariance_7005_lh | 0.003 | 3.20E-02 |
| original_shape_Flatness_7010_lh | original_glszm_ZoneVariance_7009_lh | 0.004 | 6.81E-01 |
| original_gldm_SmallDependenceEmphasis_7001_lh | original_firstorder_Skewness_7010_lh | 0.005 | 6.92E-01 |
| original_gldm_SmallDependenceEmphasis_233_lh | diagnostics_Image-original_Mean_7006_lh | 0.006 | 3.96E-01 |

## MCI vs AD

To identify the most predictive feature subset for differentiating Alzheimer's disease AD from MCI, we assessed the performance of the MLP classifier using four metrics: ROC AUC, Accuracy, Specificity, and Sensitivity.

The analysis began with LASSO feature selection, where the top 50 features were retained based on the absolute values of their non-zero regression coefficients. This ranking reflects the most informative features according to LASSO's embedded regularization. We then employed a rank-based incremental addition approach, in which features were sorted by their LASSO coefficient magnitudes and added one by one to the MLP classifier. For each subset size, the model was evaluated using stratified cross-validation.

The analysis revealed that the optimal feature subset consisted of 33 features, achieving an MLP classification performance of ROC AUC: 0.861, Accuracy: 0.806, Specificity: 0.821, Sensitivity: 0.788, as presented in Figure 4.


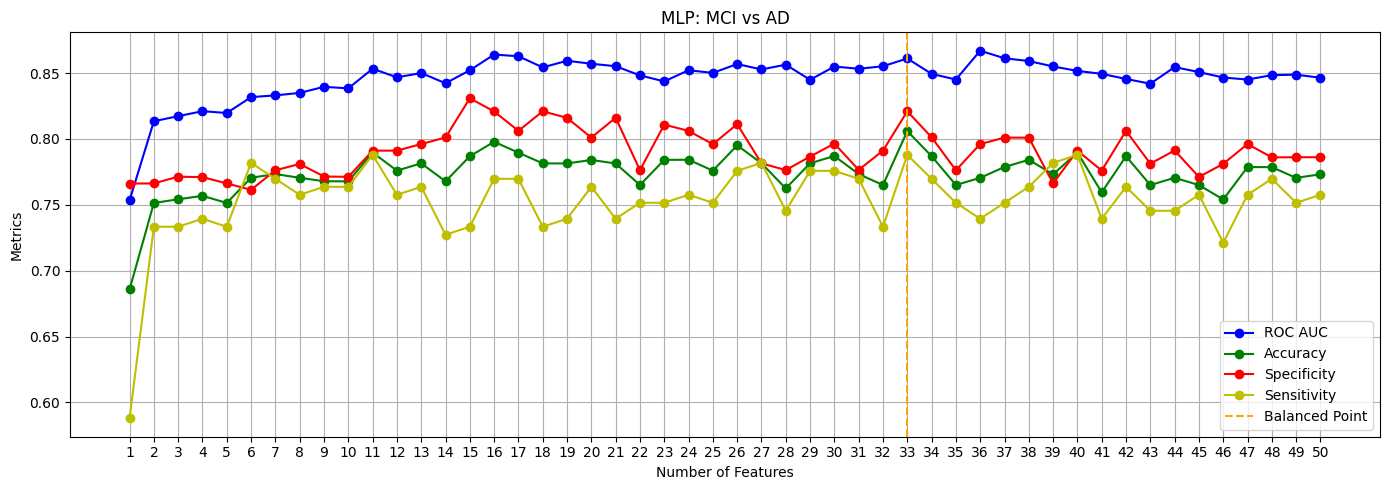


**Fig. 4.** Performance of MLP Classifier with LASSO-Selected Features for MCI vs. AD Discrimination

To enhance model interpretability and reduce redundancy, Pearson correlation analysis was conducted on the top 33 selected features to identify those with minimal interdependence (Figure 5).

Highly correlated feature pairs were excluded to mitigate multicollinearity and retain only features offering distinct contributions to the classifier. From this analysis, the feature pairs with the lowest absolute correlation coefficients were selected, reflecting weak or negligible relationships. We further validated these findings using the Spearman rank correlation test, confirming the statistical insignificance of these low correlations. The resulting Table 6 presents the selected feature pairs, their correlation coefficients, and corresponding p-values, forming a solid basis for an optimized feature set.


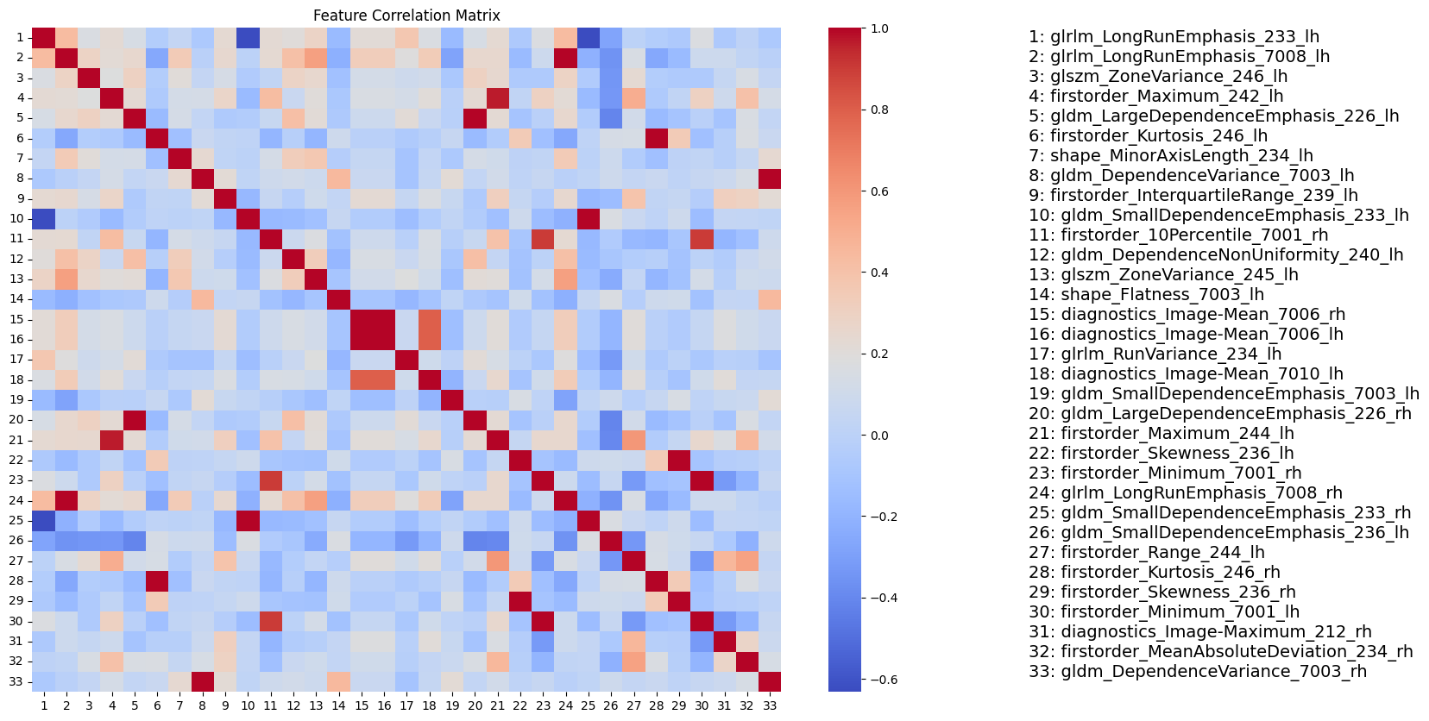


**Fig. 5.** Feature correlation matrix for the group of features with the highest accuracy in discriminating MCI from AD

**Table 6.** Highly uncorrelated feature pairs for distinguishing MCI vs AD

| Uncorrelated Features (Feature Name, FS ID, left/right hand) | | Correlation | P-Value |
| --- | --- | --- | --- |
| original_glrlm_RunVariance_234_lh | original_firstorder_Skewness_236_lh | 0.001 | 5.81E-01 |
| original_gldm_SmallDependenceEmphasis_233_lh | original_glrlm_LongRunEmphasis_7008_lh | 0.002 | 6.72E-01 |
| original_firstorder_Range_244_lh | original_glrlm_LongRunEmphasis_233_lh | 0.002 | 6.55E-01 |
| original_gldm_DependenceNonUniformity_240_lh | original_firstorder_Minimum_7001_lh | 0.003 | 7.80E-01 |
| original_firstorder_MeanAbsoluteDeviation_234_rh | original_glrlm_LongRunEmphasis_233_lh | 0.004 | 3.99E-01 |
| original_gldm_SmallDependenceEmphasis_7003_lh | original_firstorder_Maximum_242_lh | 0.005 | 8.00E-01 |
| original_glrlm_LongRunEmphasis_7008_lh | original_gldm_DependenceVariance_7003_lh | 0.006 | 7.80E-01 |
| original_glrlm_LongRunEmphasis_7008_rh | original_gldm_DependenceVariance_7003_lh | 0.007 | 7.80E-01 |
| original_firstorder_Skewness_236_rh | original_shape_MinorAxisLength_234_lh | 0.008 | 8.05E-01 |
| original_glrlm_RunVariance_234_lh | original_gldm_SmallDependenceEmphasis_7003_lh | 0.009 | 2.09E-01 |

## CN vs. MCI

The comparative analysis of CN vs. MCI classification was conducted to identify the most predictive feature subset using the MLP classifier. The process began with feature selection using LASSO regression, from which the top 50 features were retained based on the absolute magnitude of their non-zero coefficients, indicating strong predictive relevance.

Subsequently, a rank-based incremental strategy was used where features were sorted by descending LASSO coefficient magnitude and added one by one to construct feature subsets. For each subset, metrics such as ROC AUC, accuracy, specificity, and sensitivity were computed. An MLP model was trained for each subset using stratified cross-validation to ensure reliable estimation.

The objective was to identify the feature subset that achieved the highest average of ROC AUC and accuracy. Figure 6 illustrates the performance of the MLP classifier for various feature subsets. The optimal subset for CN vs. MCI classification comprised 35 features, achieving an ROC AUC of 0.803, accuracy of 0.731, specificity of 0.735, and sensitivity of 0.726. This configuration was selected as the optimal point based on the average ROC AUC and accuracy, highlighting the effectiveness of the subset in distinguishing between CN and MCI subjects.

To further refine the model and achieve a more interpretable feature set, Pearson correlation analysis was applied to the top selected features (Figure 7). This step aimed to remove highly correlated features to minimize redundancy and ensure each remaining feature contributed distinct information to the classification task. By focusing on uncorrelated features, we minimized overlap in the information represented by the selected variables.


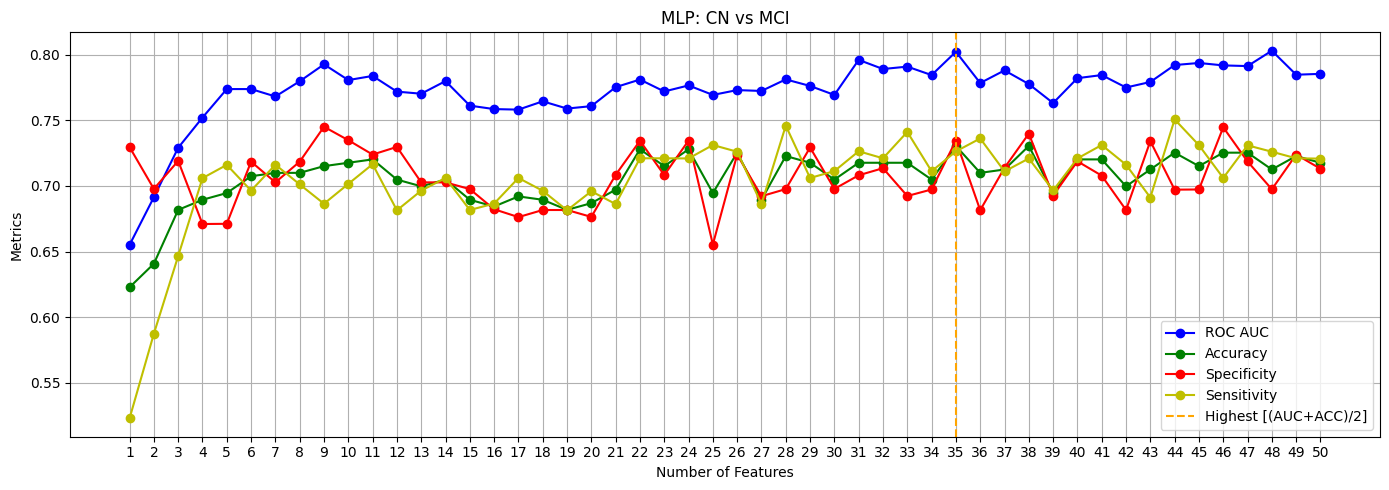


**Fig. 6.** Performance of MLP Classifier with LASSO-Selected Features for CN vs. MCI Discrimination

To identify the most diverse and representative subset, feature pairs with the lowest absolute correlation coefficients were selected based on Pearson correlation analysis. Correlations were considered negligible when the absolute Pearson r was low (e.g., |r| < 0.01), indicating independent predictive signals. The statistical significance of these correlations was validated using the Spearman rank correlation test, ensuring the robustness of the feature selection process (Table 7).

**Table 7.** Highly uncorrelated feature pairs for distinguishing CN vs MCI

| Uncorrelated Features (Feature Name, FS ID, left/right hand) | | Correlation | P-Value |
| --- | --- | --- | --- |
| original_firstorder_Skewness_236_lh | original_gldm_DependenceVariance_7001_lh | 0 | 0.706 |
| original_firstorder_Range_7007_lh | original_shape_Maximum2DDiameterColumn_7005_lh | 0.001 | 0.7609 |
| original_firstorder_InterquartileRange_211_rh | original_shape_Elongation_242_lh | 0.002 | 0.7156 |
| original_firstorder_InterquartileRange_211_lh | original_shape_Elongation_242_lh | 0.002 | 0.7156 |
| original_firstorder_Kurtosis_215_rh | original_firstorder_Range_7007_lh | 0.002 | 0.5728 |
| original_gldm_DependenceNonUniformityNormalized  _236_lh | original_firstorder_RobustMeanAbsoluteDeviation  _7008_lh | 0.002 | 0.7097 |
| original_firstorder_Minimum_241_lh | original_firstorder_Minimum_7001_lh | 0.002 | 0.5246 |
| diagnostics_Image-original_Minimum_7006_lh | original_firstorder_Variance_239_lh | 0.002 | 0.8809 |
| original_firstorder_InterquartileRange_211_rh | original_glrlm_RunVariance_226_lh | 0.003 | 0.7854 |
| original_glrlm_RunVariance_226_lh | original_firstorder_InterquartileRange_211_lh | 0.003 | 0.7854 |
| original_glszm_LargeAreaEmphasis_246_lh | original_glszm_SizeZoneNonUniformity_7005_lh | 0.003 | 0.3579 |
| original_firstorder_Variance_239_lh | original_shape_Maximum2DDiameterSlice_212_lh | 0.004 | 0.5479 |


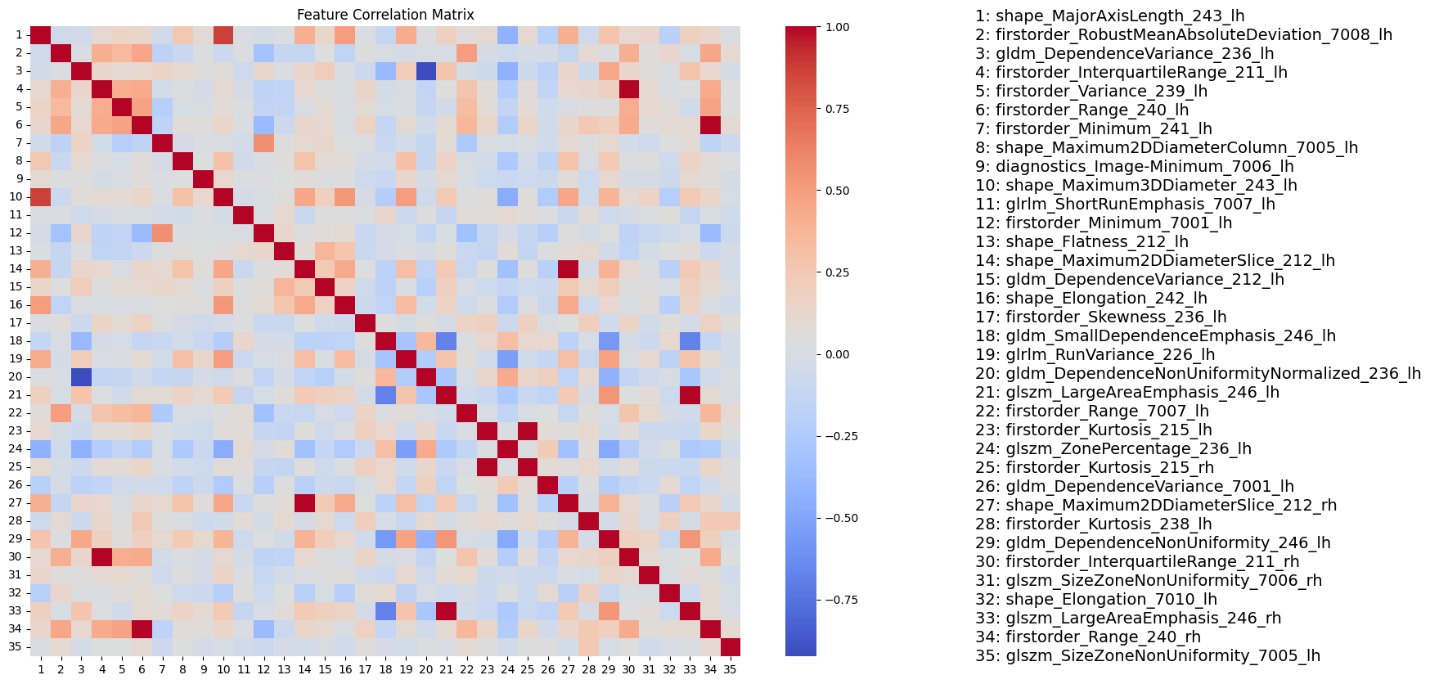


**Fig. 7.** Feature correlation matrix for the group of features with the highest accuracy in discriminating CN from MCI
